# Impact of multiple sclerosis on mental health: A Cross-Sectional Study

**DOI:** 10.1192/j.eurpsy.2023.1923

**Published:** 2023-07-19

**Authors:** S. Chemingui, M. Mersni, I. Yousfi, N. Mechergui, D. Brahim, G. Bahri, H. Ben Said, I. Youssef, S. Ernez, N. Ladhari

**Affiliations:** Department of Occupational Medicine, Charles Nicolle Hospital of Tunis, Tunis, Tunisia

## Abstract

**Introduction:**

When we think of multiple sclerosis (MS), we usually talk about the sensory and motor symptoms of the disease and their impact on the functioning of the individual affected. However, this disability can lead to a wide range of symptoms, including psychological and cognitive manifestations that also have a significant impact on the quality of life of patients

**Objectives:**

To estimate the incidence of psychiatric disorders in patients with MS.

**Methods:**

A cross-sectional descriptive study that interested MS patients referred to the occupational pathology consultation of the Charles Nicolle Hospital, during the period from July 1, 2020, to September 30, 2022. The data collected concerned the characteristics of the disease. The detection of psychiatric disorders was studied through a validated self-questionnaire GHQ-12 (General Health Questionnaire).

**Results:**

The study population consisted of 26 cases. The average age was 38 ± 9 years. A predominance of females was noted in 77% of cases. Eight patients (31%) were smokers. Nine cases (47%) had a relapsing-remitting form and six cases (32%) had a primary progressive form. All patients were on disease-modifying therapy. The average duration of the disease was 6 ± 3 years. The average duration of work during the illness was 4 years [one year-12 years]. The average duration of work stoppage in the last 12 months of activity was 63 days [2-240 days], of which 54% was long-term sick leave. The mean GHQ-12 score was 4.38 [0-10]. Twenty patients (77%) had psychological disorders.

**Conclusions:**

This study shows the high frequency of psychiatric disorders in our MS patients. The role of the neuropsychologist is therefore often crucial in the care of these patients.

**Disclosure of Interest:**

None Declared

